# Ploidy-Dependent Effects of Light Stress on the Mode of Reproduction in the *Ranunculus auricomus* Complex (Ranunculaceae)

**DOI:** 10.3389/fpls.2020.00104

**Published:** 2020-02-20

**Authors:** Fuad Bahrul Ulum, Camila Costa Castro, Elvira Hörandl

**Affiliations:** ^1^Department of Systematics, Biodiversity and Evolution of Plants, Albrecht-von-Haller Institute for Plant Sciences, University of Göttingen, Göttingen, Germany; ^2^Biology Department, Faculty of Mathematics and Sciences, Jember University, Jember, Indonesia

**Keywords:** apomixis, single seed ﬂow cytometric seed screening, light stress, meiosis, pollen, polyploidy, Ranunculus, seed formation

## Abstract

Polyploidy in angiosperms is an influential factor to trigger apomixis, the reproduction of asexual seeds. Apomixis is usually facultative, which means that both sexual and apomictic seeds can be formed by the same plant. Environmental abiotic stress, e.g. light stress, can change the frequency of apomixis. Previous work suggested effects of stress treatments on meiosis and megasporogenesis. We hypothesized that polyploidy would alter the stress response and hence reproductive phenotypes of different cytotypes. The main aims of this research were to explore with prolonged photoperiods, whether polyploidy alters proportions of sexual ovule and sexual seed formation under light stress conditions. We used three facultative apomictic, pseudogamous cytotypes of the *Ranunculus auricomus* complex (diploid, tetraploid, and hexaploid). Stress treatments were applied by extended light periods (16.5 h) and control (10 h) in climate growth chambers. Proportions of apomeiotic vs. meiotic development in the ovule were evaluated with clearing methods, and mode of seed formation was examined by single seed ﬂow cytometric seed screening (ssFCSS). We further studied pollen stainability to understand effects of pollen quality on seed formation. Results revealed that under extended photoperiod, all cytotypes produced significantly more sexual ovules than in the control, with strongest effects on diploids. The stress treatment affected neither the frequency of seed set nor the proportion of sexual seeds nor pollen quality. Successful seed formation appears to be dependent on balanced maternal: paternal genome contributions. Diploid cytotypes had mostly sexual seed formation, while polyploid cytotypes formed predominantly apomictic seeds. Pollen quality was in hexaploids better than in diploids and tetraploids. These findings confirm our hypothesis that megasporogenesis is triggered by light stress treatments. Comparisons of cytotypes support the hypothesis that ovule development in polyploid plants is less sensitive to prolonged photoperiods and responds to a lesser extent with sexual ovule formation. Polyploids may better buffer environmental stress, which releases the potential for aposporous ovule development from somatic cells, and may facilitate the establishment of apomictic seed formation.

## Introduction

Polyploidy is a heritable trait of obtaining more than two sets of chromosomes in the nuclei (Comai, 2005). A polyploid arises either from intraspecific genome duplication (autopolyploidy) or the merging of the genome of distinct species through hybridization and subsequent genome duplication (allopolyploidy) (Grant, 1981). Polyploidy is quite common in flowering plants, estimated to occur in more than 50% of species (Soltis et al., 2015) and is considered as a major factor in plant evolution (Soltis et al., 2014). Even though polyploidy is potentially obstructed by several disadvantages, e.g., disruption effects of structural enlargement of nuclei, side effects of aneuploidy, and epigenetic mutation, it also provides advantages such as heterosis, gene redundancy, and novel gene combinations. Heterosis favors polyploids that are more vigorous than their diploid progenitors, while gene redundancy protects polyploids from the deleterious effect of mutation (Comai, 2005).

Polyploidisation, with higher DNA content, increases the cell size and promotes diversity of the genome, transcriptome, and metabolome. These improvements imply a greater resistance to environmental change (Schoenfelder and Fox, 2015). Several studies reported a better adaptivity of polyploid plants to abiotic stress conditions, such as salt (Chao et al., 2013), drought (del Pozo and Ramirez-Parra, 2014; Martínez et al., 2018), drought and heat stress (Godfree et al., 2017), cold (Klatt et al., 2018), and light (Coate et al., 2013). The better stress response and adaptation of polyploids to abiotic conditions are probably under epigenetic control (del Pozo and Ramirez-Parra, 2014). Polyploidy changes the methylation profile under stressful environments, as reported, e.g. for *Brassica napus* after drought (Jiang et al., 2019).

Notably, stress conditions can also influence mode of reproduction, especially apomixis, the asexual reproduction *via* seed (Nogler, 1984a). Apomixis is widespread in angiosperms (Hojsgaard et al., 2014b), and occurs most frequently in polyploid cytotypes, but occasionally also in diploids (Grant, 1981; Carman, 1997; Hojsgaard and Hörandl, 2019). Gametophytic apomixis, the form of interest here, involves formation of an unreduced embryo sac from an unreduced megaspore *via* meiotic restitution of the megaspore mother cell (diplospory) or from a somatic cell of the nucellus tissue (apospory) (Asker and Jerling, 1992; Koltunow and Grossniklaus, 2003). Functional seed development through gametophytic apomixis involves three components: (1) apomeiosis (formation of unreduced embryo sac); (2) parthenogenesis (embryo development without fertilization of egg cell); and (3) functional endosperm development with male genome contributions from the pollen (pseudogamously) or independent from pollen (autonomously) (Nogler, 1984a). Male development is usually meiotic, but microsporogenesis is often disturbed, and hence final pollen quality is often strongly reduced (Asker and Jerling, 1992; Izmaiłow, 1996; Hörandl et al., 1997; Mráz et al., 2009). Apomixis is heritable (Ozias-Akins and van Dijk, 2007), and under genetic and epigenetic control (Grimanelli, 2012; Hand and Koltunow, 2014). Natural apomixis is frequently facultative, which means that the plant produces sexual and asexual seeds within one generation, often within the same flower or inflorescence (Bicknell et al., 2003; Aliyu et al., 2010; Cosendai and Hörandl, 2010; Hojsgaard et al., 2013; Schinkel et al., 2016).

Alternation of frequencies of asexual vs. sexual reproduction was observed under abiotic stress conditions, e.g. temperature, drought stress, salt stress, and photoperiod in many different genera (Evans and Knox, 1969; Saran and de Wet, 1976; Quarin, 1986; Gounaris et al., 1991; Klatt et al., 2016; Rodrigo et al., 2017; Klatt et al., 2018). Such a condition-dependent sex is also known from other asexual eukaryotes (Ram and Hadany, 2016). Abiotic stress leads to the accumulation of ROS (Reactive oxygen species) in plant tissues, which triggers oxidative damage, but also can initiate various epigenetic, genetic and hormonal signaling pathways for plant development (Halliwell, 2006; Foyer and Noctor, 2009; Huang et al., 2019). In the germline precursor cells, oxidative stress may increase the level of DNA double-strand breaks (DSBs) as initiator of meiosis. Here meiosis could act as DNA repair system (Hörandl and Hadacek, 2013). The above-mentioned studies on condition-dependent sex in plants support this hypothesis. In polyploids, however, an improved tolerance of stress conditions might decrease the stimulus for meiosis, and consequently trigger the alternative asexual development (Hörandl and Hadacek, 2013). However, a putative differential response of cytotypes to stress conditions with respect to mode of reproduction was so far not investigated.

We use here as a model system three cytotypes of the *Ranunculus auricomus* complex, a Eurasian polyploid complex with facultative, aposporous and pseudogamous apomixis (Nogler, 1984b; Hojsgaard et al., 2014a). In Central Europe, the *R. auricomus* complex comprises three closely related and genetically similar sexual progenitor species, and polyploid apomictic hybrids of these taxa (Hörandl et al., 2009; Hodač et al., 2014). One of the hexaploid hybrids (*R. carpaticola* x *cassubicifolius*) with facultative apomixis (Hojsgaard et al., 2014a) was used previously for testing the response to light stress. This previous experiment using extended photoperiod enhanced sexual megaspore formation in these hexaploid *R. auricomus* clones concomitant with oxidative stress (Klatt et al., 2016). In our study, we test the hypothesis that with the light stress treatment, diploids would respond more intensively to stress conditions with higher frequencies of sexual development than higher ploidy levels. Here we extend the treatment of (Klatt et al., 2016) to diploid, lower polyploid (tetraploid), and the same hexaploid plants to observe effects on mode of reproduction in different ploidy levels. To simulate the effect of extended photoperiod on the components of gametophytic apomixis, we study here two developmental steps, namely ovule formation, and seed formation. Since microsporogenesis is meiotic without an alternative asexual developmental pathway, we focus here on pollen quality as a possible factor for successful seed formation. The main aims of this research are to explore with light stress treatments whether ploidy level alters stress response with respect to mode of reproduction, and whether stress response correlates positively to sexual megaspore formation and/or proportions of sexual seed formation.

## Materials and Methods

### Plant Material

We used for the extended photoperiod experiment facultative apomictic plants of the *Ranunculus auricomus* complex from three different cytotypes. These cytotypes are hybrids that originated from three Central European parental species (*R. cassubicifolius*, *R. carpaticola*, and *R. notabilis*). The diploid plants were synthetic F2 hybrids of *R. carpaticola x notabilis* and represent sister or sibling individuals from two parental lines; see details of crossing design in Barke et al. (2018). We used these plants because natural diploid apomicts are not known for the *R. auricomus* complex. The tetraploids were garden offspring of *Ranunculus variabilis*, which is a putative natural allopolyploid of the *R. carpaticola/cassubicifolius* lineage and the *R. notabilis* lineage, and occurs sympatrically with the parental species in Central Europe (Hodač et al., 2014). The hexaploids were garden offspring of *Ranunculus carpaticola x cassubicifolius*, the same plants as used by Klatt et al. (2016). Hence, all cytotypes are hybrids, and they share the genetic background of closely related parental species (Hörandl et al., 2009). Since the parental taxa and the natural hybrids occur all in the same geographical area and altitudinal zone (Hörandl et al., 2009), we can also assume that they are all pre-adapted to the same natural light conditions. The ploidy level of tetraploids was ascertained using flow cytometry following methods of (Klatt et al., 2016). A list of materials with an identity number and ploidy levels is given in the Appendix (**Supplementary Table 1**). Plants were cultivated in the old botanical garden of the University of Goettingen from summer to winter for exposure to natural conditions, to stimulate the flower initiation.

### Growth Chamber Setup

The plants were moved into the climate growth chamber when sprouting at the beginning of the spring season. We run experiments for 2 years to get a more complete sampling. The 1st year experiment was started from the first week of March 2017; the 2nd year was started from first February 2018. A total of c. 25 plants from each cytotype were grown with 10-h photoperiod (control) and 16 plus 0.5-h photoperiod (stress treatment) following (Klatt et al., 2016). Temperature setup and relative humidity were kept stable at 18°C and 60%, respectively. The light intensity was measured with a photometer (3415F Quantum Light Meter, Spectrum Technologies, Inc, Plainfield, USA) as photoactive radiation (PAR) c. 250 µmol m^-2^ s^-2^ (measured at shoot tips).

### Plant Genotyping

Genotyping by Simple sequence repeats (SSRs) was applied to verify the plant's clonality and the relationships of cytotypes. We conducted SSRs only to tetraploid plants following methods by (Klatt et al., 2016). The SSR data for the other two cytotypes were derived from (Barke et al., 2018) for diploids and (Klatt et al., 2016) for hexaploids. Genomic DNA was performed by extracting dried leaf samples using Invisorb^®^ Spin Plat Mini Kit (Qiagen, Hilden, Germany) according to the manufacturer's protocol. Multiplex Polymerase Chain reaction (PCR) was conducted at 25 µl volumes, containing 1 µl template DNA, 12.5 Roti^®^-Pol TaqS Master mix (Carl Roth GmbH + Co. KG, Karlsruhe, Germany), 1 µl Forward Primer, 1 µl Reverse Primer, 0.125 µl MgCl_2_, 1 µl CAG-Primer (FAM or HEX labeled). PCR reactions were run in a BIORAT™ Thermal Cycler. PRC machine setting was: 94°C for 10 min, then 14 x (denaturation at 94°C for 60 s, annealing at 62°C+ 0.5°C per cycle for 90 s, extension at 72°C for 60 s), followed subsequently by 35 x (denaturation at 94°C for 30 s, annealing at 55°C for 30 s and extension at 72°C for 30 s), last extension step at 72°C for 60 s and final storage conditions at 4°C. PCR samples were adjusted before 85 µl formamide (HiDi) was added. This mixture was run in an automatic capillarity sequencer Genetic Analyzer 3130 (Applied Biosystems, Forster City, CA, USA) using Gene Scan 500 Rox (Applied Biosystems) as size standard after a denaturing pretreatment for 3 min at 92°C. Scoring of the electropherograms was done using Genemarker V2.4.2 (SoftGenetics LLC, State College, PA, USA) and exported as a binary matrix presence/absence of alleles to characterize multilocus genotypes. We applied Neighbour-joining analysis based on Jaccard similarity index in FAMD to test the SSR profiles (Schlüter and Harris, 2006). Branch support values were derived from the majority consensus tree from 1000 bootstrap replicates. FigTree v1.4.2 (Rambaut, 2007) visualized the result.

### Female Development

Development of embryo sacs was already previously characterized within the *R. auricomus* complex on both apomictic and sexual species and is quite uniform (Nogler, 1984a; Hojsgaard et al., 2014a; Klatt et al., 2016; Barke et al., 2018): the megaspore mother cell differentiates near the micropyle and undergoes meiosis, resulting in a megaspore tetrad. In sexual development only the chalazal megaspore develops further, and produces after three mitotic divisions a typical 7-celled, 8-nucleate *Polygonum* type embryo sac (with three antipodals, a binucleate central cell, two synergids, and one egg cell). Apomictic development is characterized by enlargement of a somatic cell in the nucellus which emerges in parallel and aside the megaspore tetrad, and continues embryo sac development into an unreduced *Polygonum* type embryo sac, whereas all megaspores abort. Embryological analysis of the female development was made at the end of sporogenesis and the beginning of gametogenesis, following Hojsgaard et al., 2014a and Barke et al. (2018). *R. variabilis*, the only taxon that was analyzed for the first time here, did not show any deviations in timing or type of development. Flower buds were fixed at Formalin: acetic acid: ethanol: dH_2_O (2: 1: 10: 3.5) (FAA) for 48 h, and stored in 75% ethanol (Hojsgaard et al., 2014a). The flower bud was treated by dehydrating in four steps of 30 min incubation in 1 ml of 70%, 95%, and 100% (two times). Then the flower buds were treated by clearing method in five steps of 30 min in 300 µl of upgrading series of methyl salicylate diluted in ethanol [25%, 50%, 70%, 85%, and 100%; (Young et al., 1979)]. The perianth of selected flower buds was removed, ovaries were dissected and mounted in methyl salicylate on glass slides. Female sporogenesis and early stages of sexual or aposporous gametophyte development were analysed with differential interface contrast (DIC) in a light transmission microscope (Leica DM5500B with DFC 450 Camera, LAS V41 software, Leica Microsystems, Wetzlar, Germany). The determination of sexual and asexual ovules was made by the absence or presence of aposporous initial cells (AIC), respectively (van Baarlen et al., 2002). We excluded ovules with unclear structure and aborted ones. We only considered the data from a plant that had a minimum of five observable ovules. Additional data from (Klatt et al., 2016) were added to increase the N value for the hexaploid cytotype.

### Seed Set

After we collected the sample for embryological analysis, the remaining flowers were then manually pollinated to increase fertilization rates. In fruiting stages, we bagged a minimum of five peduncles with collective fruits with porous plastic bags to avoid seed loss. We harvested the mature collective fruits and evaluated the proportion of well-developed seeds (seed-set percentage) among ploidies per flower on individual according to Hörandl (2008). Well-developed seeds were stored at room temperature and were used for reproductive pathway analysis.

### Reproductive Pathway of Seed Formation

The reproductive pathway was evaluated by single seed flow cytometric seed screening (ssFCSS) (Matzk et al., 2000). Two steel balls grounded a single seed (Ø 4 mm) in a 2 ml Eppendorf tube in a TissueLyzer II (Qiagen, Hilden, Germany; 30 Hz s-1 for 7 s). Nuclear isolation and staining were attained in two steps using Otto buffers (Otto, 1990). In the first step, nuclear isolation, 200 µl Otto I buffer (0.1 M citric acid monohydrate, 0.5% v/v Tween 20) was added and hand shacked with the ground material for 30 s. The solution was then filtered (30 µm mesh, Celltrics^®^ Münster, Germany) into plastic tubes (3.5 ml, 55 mm x 12 mm, Sarstedt, Nümbrecht, Germany). In the second step, staining, 800 µl otto II buffer [0.4 M Na_2_HPO_2_, ddH_2_O and charged with 3 ng/ml 4',6-diamidinophenyl-indole (Sigma-Aldrich, Munich, Germany)] was added to the filtrate, and the solution was measured directly in Flow cytometer (CyFlow^®^ Ploidy Analyser (Sysmex Partec GmbH, Görlitz, Germany) in the Blue fluorescence (UV LED, gain 365). Histograms were analyzed using CyView™ V.1.6 software (Partec GmbH). The coefficients of variation were less than 8%. The ploidy levels of embryo and endosperm were determined, and peak indices (PI) (mean peak value of the embryo compared to the mean peak of endosperm) were assessed (**Supplementary Figure 5**). For a *Polygonum* type embryo sac with two polar nuclei, the peak index for a sexual seed is c. 1.5, while for asexual seeds it can be 2.0, 2.5, or 3.0, depending on the contribution of pollen nuclei to endosperm formation. We observed the following developmental pathways: Sexual, pseudogamous apomixis, autonomous apomixis, and B_III_-hybrids (Hojsgaard and Hörandl, 2019). B_III_-hybrids arise from an unreduced embryo sac, whereby egg cell and polar nuclei were fertilized. The B_III_-hybrids were excluded for the determination of the proportion of sexual seeds since this mode of reproduction is intermediate between sexual and asexual seed formation.

### Pollen Stainability

Pollen stainability was determined on a minimum of 500 pollen grains per plant from all cytotypes in both chambers by using 10% Lugol's iodine (I_2_KI) solution, following methods by (Schinkel et al., 2017). The stainability of starch content was used as an indicator of viable pollen under a light microscope (LEICA DM5500B with DFC 450 C camera, LAS V41 software, Leica Microsystems, Wetzlar, Germany) at 400x magnification. The viable pollen grains were indicated by black-stained color, but brownish, reddish, and translucent (empty) pollen was counted as non-viable.

### Statistical Analyses

All data were tested for their normality distribution by Kolmogorov-Smirnov and Shapiro-Wisk test and for their homogeneity of variance with the Levene test. Female development, seed set, reproduction pathway of seed formation, and pollen viability were determined per flower as a percentage and subsequently averaged per plants. The percentage of data were arcsine transformed before statistical analysis. We tested the influence of treatment on mean sexual ovules and seed set among ploidies with General Linear Model (GLM) univariate (Two-way ANOVA) for completely randomized factorial design model, and means were compared according to the least significant difference (LSD) test at 0.05 probability level (p-value < 0.05). Tukey HSD was performed to the means of sexual ovules to determine the main factors. Nonparametric Kruskal-Wallis and Mann-Whitney U-test were applied to test the influence of treatment on sexual seed formation per ploidy. Boxplots were plotted with untransformed percentage values and show the 25^th,^ and 75^th^ percentile ranges as a box, and the median as a black line: circles are outliers; asterisks are extreme values. All statistical analyses were performed with IBM SPSS Statistic 25 (IBM Deutschland GmbH).

## Results

### Female Development

**Figure 1:**
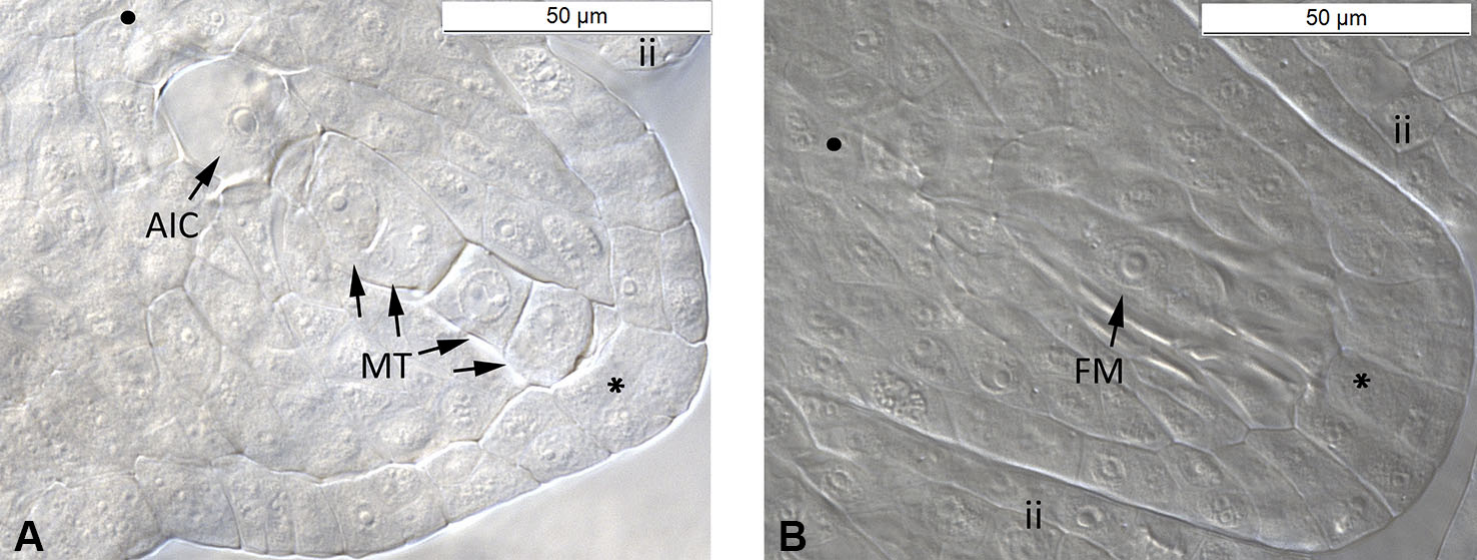
Megasporogenesis of *R. variabilis* plants. **(A)** Asexual ovule during megaspore formation. The germline with megasporocyte tetrad and one aposporous initial cell near the chalazal pole is shown. **(B)** Sexual ovule during functional megaspore formation. Only one cell near the chalazal pole survived and developed into a functional megaspore whereas the other three cells are aborted. Plant individual: **(A)** LH1406030B4-7 (Tetraploid); **(B)** LH1406030B4-19 (Tetraploid). AIC, Aposporous Initial Cell; FM, Functional Megaspore; ii, inner integument; MT, Megaspore Tetrad; SY, Synergid; •, chalazal pole; *, micropylar pole. Scale bar: 50 µm.

The ovule development of all three cytotypes of the *R. auricomus* complex showed the same pattern of a typical *Polygonum* type embryo sac (**Supplementary Figures 1**–**4**). We had observed 6,505 ovules (c. 18 ovules per flower bud) among cytotypes at megasporogenesis and early megagametogenesis. At this stage, sexual and asexual ovules can be discriminated (**Supplementary Figure 4**). At the megasporogenesis stage, a meiotic division of a megaspore mother cell produced four cells, i.e. a megaspore tetrad. During the next step, three cells aborted, and only the chalazal cell remained as functional megaspore. At megagametogenesis stage, the functional megaspore enlarged with the presence of vacuoles and continued with three nuclear divisions, resulting in a total of eight nuclei. Development of sexual ovules was indicated by the absence of any aposporous initial cell (AIC) during megasporogenesis and early megagametogenesis. On the other hand, in asexual ovules, one or more AIC was observed directly near the megaspores at the chalazal pole or near to this area, but at a different optical layer (**Figure 1**).

### Effects of Ploidy, Treatment, and Combined Effect of Ploidy/Treatment to the Proportion of Female Development

**Figure 2:**
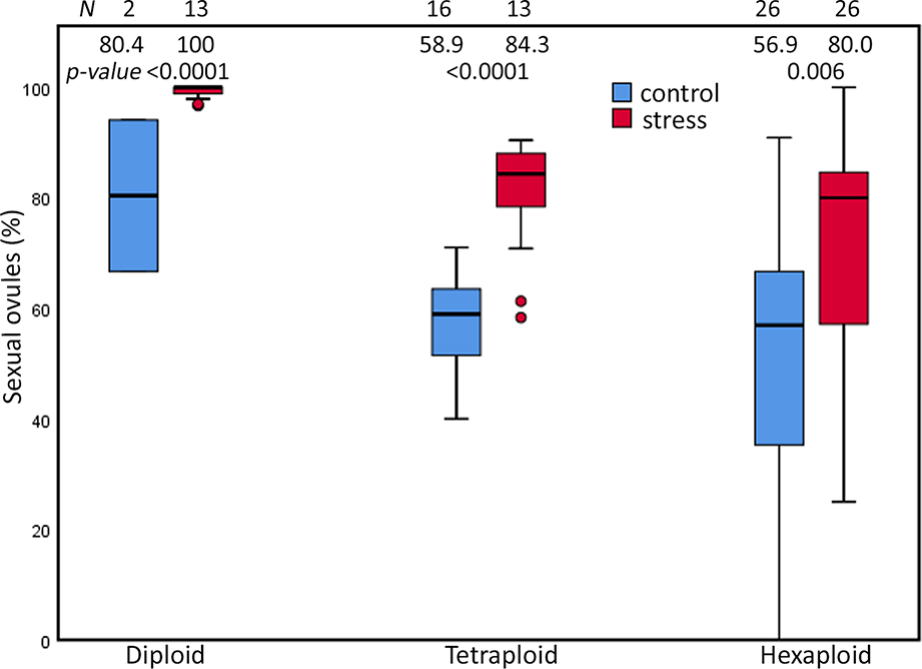
Proportions of sexual ovules in the *R. auricomus* complex plants grown in climatic chamber under prolonged photoperiod (stress) and shortened photoperiod (control). Mean values and statistical significance are given in figure. N = number of individuals. For the test statistics, see **Supplementary Table 2**.

Extended photoperiod enhanced the proportion of sexual ovules in all three cytotypes of the *R. auricomus* complex. The mean proportion of sexual ovules significantly increased from control treatment to stress treatment (80.37 (mean) ± 19.38 (sd) % to 99.26 ± 1.26%; p-value < 0.001) in diploid, (57.90 ± 8.79% to 80.29 ± 10.67%; p-value < 0.001) in tetraploids, and 52.61 ± 26.11% to 70.36 ± 20.04%; p-value = 0.006) in hexaploids (**Figure 2**). ANOVA revealed significant alterations by the main effect photoperiod (p-value < 0.001) and ploidy (p-value < 0.001), but not by the interrelationship between them (p-value = ns) (**Table 1**). Tukey HSD revealed significant differences in control treatment between diploids and hexaploids (p-value = 0.047) and in stress treatment between diploids and polyploids (p-value < 0.001) but there is neither a significant difference between tetraploids and hexaploids in the both treatments nor among diploids and tetraploid in the control treatment (p-value = ns) (**Supplementary Table 3**).

**Table 1:** P-values for the two way ANOVAs to determine the interaction effect of stress treatment and ploidy level on the proportion of sexual ovules.

Source	Type III Sum of Squares	df	Mean Square	F	Sig.
Ploidy	1.769	2	0.885	14.091	0.001
Treatment	1.529	1	1.529	24.357	0.001
Ploidy x Treatment	0.132	2	0.066	1.053	0.353

R Squared = 0.574 (Adjusted R Squared = 0.551)

### Seed Set

**Figure 3:**
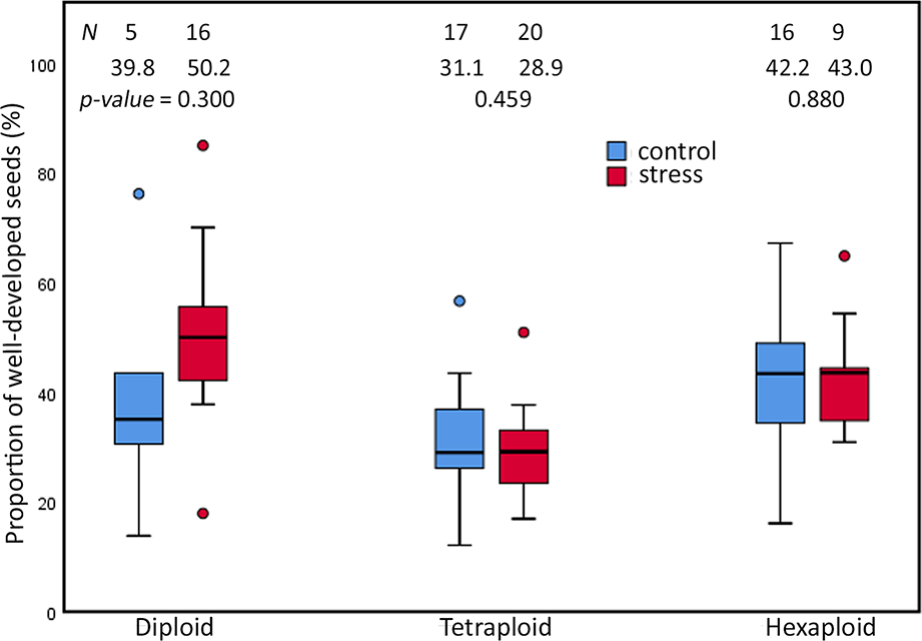
Proportions of well-developed seeds in the *R. auricomus* complex plants grown in climatic chambers under prolonged photoperiod (stress) and shortened photoperiod (control). Mean values and statistical significance are given in figure. N = number of individuals. For the test statistic, see **Supplementary Table 2**.

Extended photoperiod did not influence the proportion of well-developed seeds among cytotypes of *R. auricomus* complex. Our investigation of 83 individuals revealed no significant difference in seed set between plants grown in control and stress chamber (p-value = ns) (**Figure 3**). Diploid plants under stress treatment produced a higher mean but not significant different proportions of well-developed seeds (mean value = 50.22%) compared to control treatments (mean value = 39.84%; p-value = 0.300). Tetraploid plants under stress treatment produced a mean of 28.97% compared to a mean of 31.09% (p-value = 0.459) under control treatment. Hexaploid plants under stress treatment produced a mean of 43.04% compared to a mean of 42.17% (p-value = 0.880) under control treatment. A two-way ANOVA revealed only significant differences between the ploidies (p-value < 0.001), but neither a significant effect on treatment nor an interaction effect (p-value = ns) (**Supplementary Table 4**). Multiple comparison tests revealed that significant differences were observed between diploids and tetraploids (p-value < 0.001; Tukey HSD) and between tetraploids and hexaploids (p-value < 0.001; Tukey HSD) (**Supplementary Table 5**).

### Reproductive Pathways of Seed Formation

**Figure 4:**
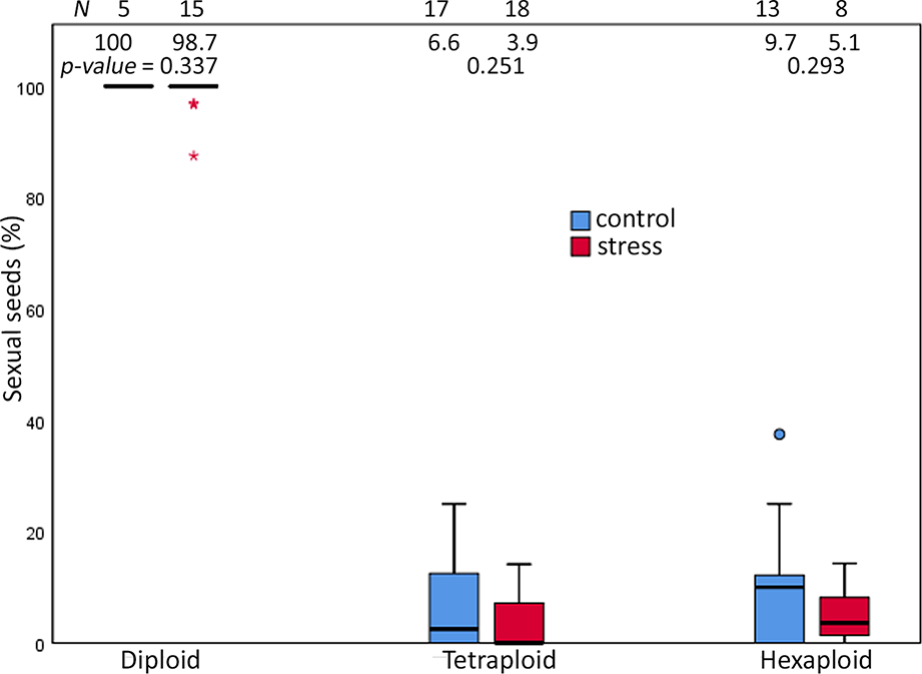
Proportions of sexual seeds in the *R. auricomus* complex plants grown in climatic chambers under prolonged photoperiod (stress) and shortened photoperiod (control). Mean values and statistical significance are given in figure. N = number of individuals. For the test statistic, see **Supplementary Table 2**.

Extended photoperiod did not enhance the proportion of sexual seeds over ploidies. The mean value of the proportion of sexual seeds was not significantly different between treatments among ploidies (p-value = ns, Mann-Whitney U-test) (**Figure 4**, **Supplementary Table 6**). Analysis of 1,468 seeds among ploidies indicated several reproductive pathways in the *R. auricomus* complex (**Table 2**). In diploid plants, the majority of seeds was formed sexually while in tetraploid and hexaploid plants, asexuality was the most frequent reproduction mode (**Figure 4**). In diploid sexual seeds, we observed the ratio of embryo to endosperm DNA content of 2C:3C, which is the indication of double fertilization between reduced egg cell with one sperm cell [1(m)+1(p)] and two polar nuclei with the other sperm cell [1(m)+1(m)+1(p)], producing a Peak Index (PI) of 1.5. A few apomictic seeds were observed (two with pseudogamous endosperm and one with autonomous endosperm) only in the stress treatment. The pseudogamous endosperm comes from the development of an unreduced embryo [2(m)] and fertilization of two polar nuclei with one or two reduced or unreduced sperm cells [2(m)+2(m)+1(p) or 2(p)], with ratios of embryo to endosperm of 2C:5C (PI = 2.5) and 2C:6C (PI = 3.0). Autonomous endosperm develops from an unreduced embryo [2(m)] and unfertilized of two polar nuclei (2Cm+2Cm) with the ratio of embryo to endosperm of 2C:4C (PI = 2.0), which is caused by the absence of paternal genome in seed development.

**Table 2:** Observed reproductive pathways of three cytotypes of the *R. auricomus* complex.

Ploidy	Reproduction mode	Genome contribution to embryo/endosperm			Sperm nuclei contribution to endosperm	PI	Number of observations (ssFCSS)	
		Embryo (Cx)	Endosperm (Cx)	Em : End			Control	Stress
Diploid	Sexual	1(m)+1(p)	2(m)+1(p)	2C:3C	1	1.5	77	282
	Apomictic	2(m)	2(m)+2(m)†	2C:4C	0	2	0	1
		2(m)	4(m)+1(p)‡	2C:5C	1	2.5	0	1
		2(m)	4(m)+1(p)+ 1(p) or 4(m)+2(p)‡	2C:6C	2 or 1	3	0	1
Tetraploid	Sexual	2(m)+2(p)	4(m)+2(p)	4C:6C	1	1.5	19	20
	Apomictic	4(m)	4(m)+4(m)†	4C:8C	0	2	0	2
		4(m)	8(m)+2(p)‡	4C:10C	1	2.5	10	24
		4(m)	8(m)+2(p)+ 2(p) or 8(m)+4(p)‡	4C:12C	2 or 1	3	196	307
		4(m)	8(m)+4(p)+ 4(p)‡	4C:16C	2	4	2	4
	B_III-_ hybrid	4(m)+2(p)	8(m)+2(p) +2(p)	6C:12C	2	2	22	3
		4(m)+2(p)	8(m)+2(p)	6C:10C	1	1.6	5	15
Hexaploid	Sexual	3(m)+3(p)	6(m)+3(p)	6C:9C	1	1.5	22	14
	Apomictic	6(m)	6(m)+6(m)†	6C:12C	0	2	5	2
		6(m)	12(m)+3(p) ‡	6C:15C	1	2.5	20	19
		6(m)	12(m)+3(p) +3(p) or 12(m)+6(p) ‡	6C:18C	2 or 1	3	246	142
		6(m)	12(m)+6(p) +6(p) ‡	6C:24C	2	4	3	3
	B_III-_ hybrid	6(m)+3(p)	12(m)+3(p)+3(p) or 12(m)+6(p)	9C:18C	2 or 1	2	1	0

†Autonomous endosperm.

‡Pseudogamous endosperm, polar nuclei were fertilized by one reduced/unreduced or two reduced/unreduced sperm nuclei.

Cx reflects ploidy based on DNA content: m, maternal genome contribution; p, paternal genome contribution; PI, peak index.

Tetraploid and hexaploid plants displayed more variation on the mode of seed reproduction. Sexual reproduction mode was present in 39 (6.2%) tetraploid seeds and 36 (7.5%) hexaploid seeds. Pseudogamous endosperm was the most frequent mode of seed formation and appeared in 543 (86.3%) tetraploid seeds and 433 (90.7%) hexaploid seeds. Generally, this mode of reproduction produced a PI value of 3.0. The less frequent forms of pseudogamous endosperm with a PI = 2.5 and PI = 4.0 originated from the contribution of one reduced sperm nucleus or two unreduced sperm nuclei. Autonomous endosperms (PI = 2.0) were the most infrequent mode of seed formation, in a total of four seeds (0.55%) from tetraploids and nine seeds (1.93%) from hexaploids. Another type of reproduction mode, i.e. partial apomixis with an unreduced egg cell fertilized by reduced pollen (B_III_-hybrid), was more frequent in tetraploid plants (45 seeds or 12.43%) compared with only one case in hexaploid plants (**Table 2**).

### Pollen Stainability

Extended photoperiod did not alter the proportion of viable pollen between treatments. The assessment through 34,348 pollen grains from 67 plants revealed no significant differences in pollen viability between plants of the same cytotype grown in both treatments (p-value = ns; see **Supplementary Figure 6**). Hexaploids produced a higher mean proportion of viable pollen (mean value = 64.6% in control treatment and 60.7% in stress treatment) compared to diploids (49.9% in control treatment and 52.9% in stress treatment) and tetraploids (50.3% in control treatment and 52.4% in stress treatment). Multiple comparison tests among ploidies revealed that the only significant differences were observed between tetraploid and hexaploid plants (p-value < 0.001; Tukey HSD; **Supplementary Table 7**).

## Discussion

Mode of reproduction in the facultative apomictic plant is influenced by abiotic stress, e.g. by light (Knox, 1967; Saran and de Wet, 1976; Quarin, 1986; Klatt et al., 2016). However, these studies compared stress and control treatments only within the same cytotype. Under the same conditions, the degree of facultative apomixis is usually related to ploidy level (Delgado et al., 2016; Kaushal et al., 2018). In this study, we presented for the first time developmental patterns among three cytotypes of the *R. auricomus* complex under stress and control conditions. We tested the hypotheses that prolonged photoperiod enhances only the first component of apomixis, i.e., apomeiotic embryo sac development, with the expectation of a buffer effect of stress in polyploids. The other two apomixis components, i.e. parthenogenesis and endosperm development, were not affected by different photoperiods.

### Effects of Ploidy, Treatment, and Combined Effect of Ploidy/Treatment to the Proportion of Female Development

Prolonged photoperiod enhanced the proportion of sexual ovules, with a greater effect on diploids but lesser effect on tetraploids and hexaploids. Enhancement on the proportion of sexual ovules after the same type of light stress had been reported before only in the hexaploid cytotype (Klatt et al., 2016). The hexaploids also formed a comparable proportion of sexual ovules under garden conditions (Hojsgaard et al., 2014a). The three cytotypes of the *R. auricomus* complex exhibited a similar mode of reproduction as the pairwise comparison of data revealed insignificant differences between ploidies in control treatments. The result of controls and also the high genetic similarity of the three cytotypes (**Supplementary Figure 7**) make it unlikely that slightly different genetic backgrounds of the cytotypes had influenced the results of our experiments. The proportion of sexual ovules of the diploid cytotype grown in the garden, ranging from 45% to 82% (Barke et al., 2018), was still within the range of our data. These plants represent recently formed synthetic F2 hybrids (Barke et al., 2018) with lower proportions of apospory than in the polyploids that already had established apomixis in the natural source populations. However, despite these more lineage-specific features, differential effects of treatments were observed in all three cytotypes in the early stages of development.

The prolonged photoperiod (16 plus 0.5 h) may have expanded the accumulation of ROS (Reactive oxygen species) in the reproductive tissue, as reported for the hexaploids based on analysis of secondary metabolite profiles (Klatt et al., 2016). Results support the hypothesis that the oxidative lesions might mobilize the meiotic DNA repair system in the megaspore mother cell and trigger meiosis and megasporogenesis (Hörandl and Hadacek, 2013). This stimulus might increase the proportion of functional megaspores as a cellular survival strategy for the germline (Rodrigo et al., 2017), as shown remarkably in our diploids. Differential genetic stress regulation of sexual and apomictic plants was also observed in seedlings of *Boechera*, and may be important for the bypass of the meiotic pathway (Shah et al., 2016).

In tetraploids and hexaploids, the oxidative stress of prolonged photoperiods might be different. This could be due to altered photosynthetic electron transport capacities (Coate et al., 2013), or to altered secondary metabolite profiles in polyploids and hybrids (Orians, 2000). We speculate that lowered oxidative stress in polyploids might not be severe enough to induce sufficient double strand breaks that would be essential for a correct processing of meiosis (Keeney et al., 2014). Consequently, meiosis and megasporogenesis might be disturbed. Failure of megasporogenesis might release aposporous initial cell (AIC) development. Cell-specific transcriptome studies on aposporous *Hieracium* subg. *Pilosella* suggested that contact and cross-talk between AICs and functional megaspores could be the trigger for mitotic development of the former and degeneration of the latter (Juranic et al., 2018). We suppose a similar interaction of AICs and megaspores in the *Ranunculus auricomus* complex as they always occur together in close neighborhood, and we observed the presence of AICs together with young (2-nucleate stage) meiotic embryo sacs but not at later stages (**Supplementary Figures 2C, D** and **Supplementary Figure 4**). The emergence of aposporous initials starts in the *Ranunculus auricomus* complex mostly at the end of megasporogenesis and is correlated to disturbance of megasporogenesis. The surviving aposporous cells grow faster than the meiotic cell and occupy the mode of megagametogenesis and seed development (Hojsgaard et al., 2014a; Barke et al., 2018). The stress only affects the megaspore but leaves apomeiosis as the surrogate for the sexual pathway (Hörandl and Hadacek, 2013). Alternatively, polyploids with more DNA content have more repair templates for the DSBs, and a higher dose of stress would be required to break the DNA (Schoenfelder and Fox, 2015). Here polyploidy might promote DNA damage tolerance under elevated stress as described (Schoenfelder and Fox, 2015) and buffers stress effects (Hörandl and Hadacek, 2013).

Environmental stress plays a role as an inhibition factor under an epigenetic mechanism that disturbs or interrupts the silencing signal of apomictic-conditioning (Rodrigo et al., 2017). At least in diploid *Ranunculus*, the treatment might strengthen a signal transduction pathway that promotes switching from apomeiosis to meiosis, as demonstrated in facultative *Boechera* after drought stress (Mateo de Arias, 2015; Gao, 2018; Carman et al., 2019). In polyploids, the whole duplication genome (WDG) provides the co-loss or co-retention condition, which maintains a constant set of miRNA for basic biological functions (Liu and Sun, 2019). Our data suggested that polyploids respond to the stress *via* homeostatic regulation in the frequency of apospory vs. megasporogenesis. The high variability of the proportions of sexual ovules among our genetically identical polyploids supports the findings of epigenetic and transcriptional control mechanisms as the background for the phenotypic expression of apospory (Grimanelli, 2012; Schmidt et al., 2014). Our result supports the hypothesis that phenotypic features of apomixis in flowering plants are strongly affected by polyploidy (Delgado et al., 2016; Kaushal et al., 2018) and subjected to epigenetic control (Rodrigo et al., 2017).

### Effects of Ploidy, Treatment, and Combined Effect of Ploidy/Treatment to the Seed Development and Mode of Reproduction

The prolonged photoperiod affected neither the frequency of seed set, the proportion of sexual seeds, nor the pollen viability. *Ranunculus auricomus* complex plants generally lose a high seed proportion compared to rates of ovule formation due to their high seed abortion rate, exceeding one-half to two-third (Izmaiłow, 1996; Hörandl, 2008; Hörandl and Temsch, 2009; Klatt et al., 2016; Barke et al., 2018). This failure on seed formation arises at early stages and during the development of endosperm tissue (Barke et al., 2018). The diploid cytotype, which generally reproduces sexually, delivers a better seed set than the higher ploidy levels. In contrast, tetraploids and hexaploids, which are predominantly facultative apomictic, showed a reversed pattern, by increasing frequencies of asexual seeds.

Pollen quality is an external factor influencing the seed set of all cytotypes. The great variation in pollen quality, as observed here, is typical for apomictic plants (Asker and Jerling, 1992). The lower quality of tetraploid pollen was concomitant with a lower seed set of the tetraploids, while the better pollen quality in diploids and hexaploids corresponded to a higher seed set in these cytotypes. For seed formation, the contribution of a male gamete to fertilize the central nuclei is the major requirement for proper endosperm development (Vinkenoog et al., 2003). The diploids keep their sexual ovules growing into sexual seeds in both treatments, while the survival of three apomictic seeds in the stress treatment represented rare exceptions from seed abortion. Similar results have been reported from the garden experiment (Barke et al., 2018). Diploid plants are sensitive to genomic imprinting deviation in the endosperm (Hörandl and Temsch, 2009; Barke et al., 2018), i.e. a 2:1 constant ratio for maternal (m) to paternal (p) genome contribution to endosperm (Spielman et al., 2003; Vinkenoog et al., 2003). The occurrences of genome imbalance in pseudogamously (4m:1p and 4m:2p) and autonomously formed seed (4m:0p) suggested that endosperm imbalance inhibited apomictic seed formation in our diploid cytotype.

On the other hand, in polyploids, the development of sexual ovules aborted to a large extent and was replaced by aposporous initials that completed megagametogenesis. Apomictic seed formation in polyploids is mainly influenced by the competitive capacity of the unreduced embryo sac formation rather than by the light regime during megagametogenesis and seed development (Hojsgaard et al., 2013; Klatt et al., 2016; Hodač et al., 2019). The surviving aposporous initials continue to develop into aposporous embryo sacs, and seeds are formed mostly *via* parthenogenesis and pseudogamous apomixis. This mode of reproduction is indicated by the parthenogenetic embryo (an unreduced egg cell develops without male gamete fusion) and pseudogamous endosperm (two unreduced polar nuclei fuses with one or two male gametes). Parthenogenesis appears mostly in our asexual polyploid seeds as a significant factor promoting unreduced gametophytes against reduced one and seed formation (Hojsgaard and Hörandl, 2019). A significant number of B_III_-hybrids in tetraploids were formed through fertilization of unreduced egg cells as partial apomixis, as it was also occasionally observed in other FCSS studies (e.g. Schinkel et al., 2016; Barke et al., 2018; Klatt et al., 2018). This B_III_-hybrid had probably an extremely long period of egg cell receptivity in this cytotype as assumed in diploid *Ranunculus* (Barke et al., 2018). Additionally, pollen-independent seed development *via* autonomous apomixis was also a rare event in polyploids. Asexual seed formation *via* pseudogamy is predominant in most apomictic plants (Mogie, 1992) as observed in our polyploids. The most common developmental pathway, however, used both sperm nuclei, or the unreduced sperm nucleus, for fertilization of polar nuclei, and hence restored the optimal 2m:1p ratio in the endosperm; these pathways result in a peak index of 3.0 in flow cytometric seed screening and represent the major proportions of apomictic seeds in both tetraploids (92%) and hexaploids (88%), see data in **Table 2**. Unbalanced genome contributions were also observed. Even though the diploids are quite sensitive to genomic imprinting, the polyploids in *Ranunculus* are more relaxed as expected (Grimanelli et al., 1997; Quarin, 1999). The current theory suggests that epigenetic mutation in polyploids creates relaxation on genomic imprinting during endosperm development (Kaushal et al., 2018). This could be the reason of higher seed set in hexaploid than in tetraploid cytotypes, similar to in hexaploid *Potentilla puberula* that had higher seed set than the tetraploids (Dobeš et al., 2018). These findings suggest the presence of a buffer effect on genomic imprinting in polyploids.

Our results suggest that the light regime only affects the proportion of sexual ovules, but the effect does not continue on the mode of seed formation. This finding supports the oxidative stress initiation hypothesis (Hörandl and Hadacek, 2013) that light stress affects only female meiosis, but has no relevance to further development. Polyploids express predominantly apospory, probably by improved mechanisms to buffer the abiotic stress, and are able to establish apomictic seed formation. These findings are in line with the general observation that apomixis mostly occurs in polyploid plants, despite the fact that the pathway can occur in diploids as well, albeit in much lower frequencies. Hence, stress resistance of polyploids may indirectly facilitate the establishment of apomixis, but is not necessarily essential for its expression, as proposed by Hojsgaard and Hörandl (2019).

## Conclusions

Three cytotypes of facultative *R*. *auricomus* complex express the alternation of proportions of asexual ovules into more sexual ovules after prolonged photoperiod. We hypothesize that light stress increases ROS formation that triggers oxidative stress. The oxidative stress might stimulate the meiotic DNA repair system in the megaspore mother cell and suppresses mitotic division, resulting in sexual ovules. The effect of prolonged photoperiod on megasporogenesis was most pronounced in diploids; the lower effect of light stress in polyploids is probably as a consequence of higher stress resistance. In polyploids, high rates of seed abortion left a lower proportion of sexual seeds, whereas in diploids the sexual pathway is still predominant. Seed formation is not influenced by environmental stress conditions, but rather depending on proper endosperm formation. Our findings shed light on the predominance of apomixis occurrence in polyploid plants.

## Data Availability Statement

The raw data supporting the conclusions of this article will be made available by the authors, without undue reservation, to any qualified researcher.

## Author Contributions

FU and EH designed research. FU performed research, analyzed and interpreted data. CC contributed to FCSS and microsatellite analysis. FU wrote the manuscript with contributions of EH.

## Funding

This project was funded by The German Research Fund DFG (DFG Hörandl Ho 4395 4-1) to EH and by the Indonesia endowment fund for education, grant no. PRJ-2369/LPDP.3/2016 to FU.

## Conflict of Interest

The authors declare that the research was conducted in the absence of any commercial or financial relationships that could be construed as a potential conflict of interest.
