# Supplementary material for: Ploidy-Dependent Effects of Light Stress on the Mode of Reproduction in the Ranunculus auricomus Complex (Ranunculaceae)
Source: Front Plant Sci. 2020 Feb 20;11:104. doi: 10.3389/fpls.2020.00104 (PMC7044147; doi:10.3389/fpls.2020.00104)
Supplement: Supplementary file 1 [file DataSheet_1.pdf]

Supplementary Material

1 Supplementary Figures

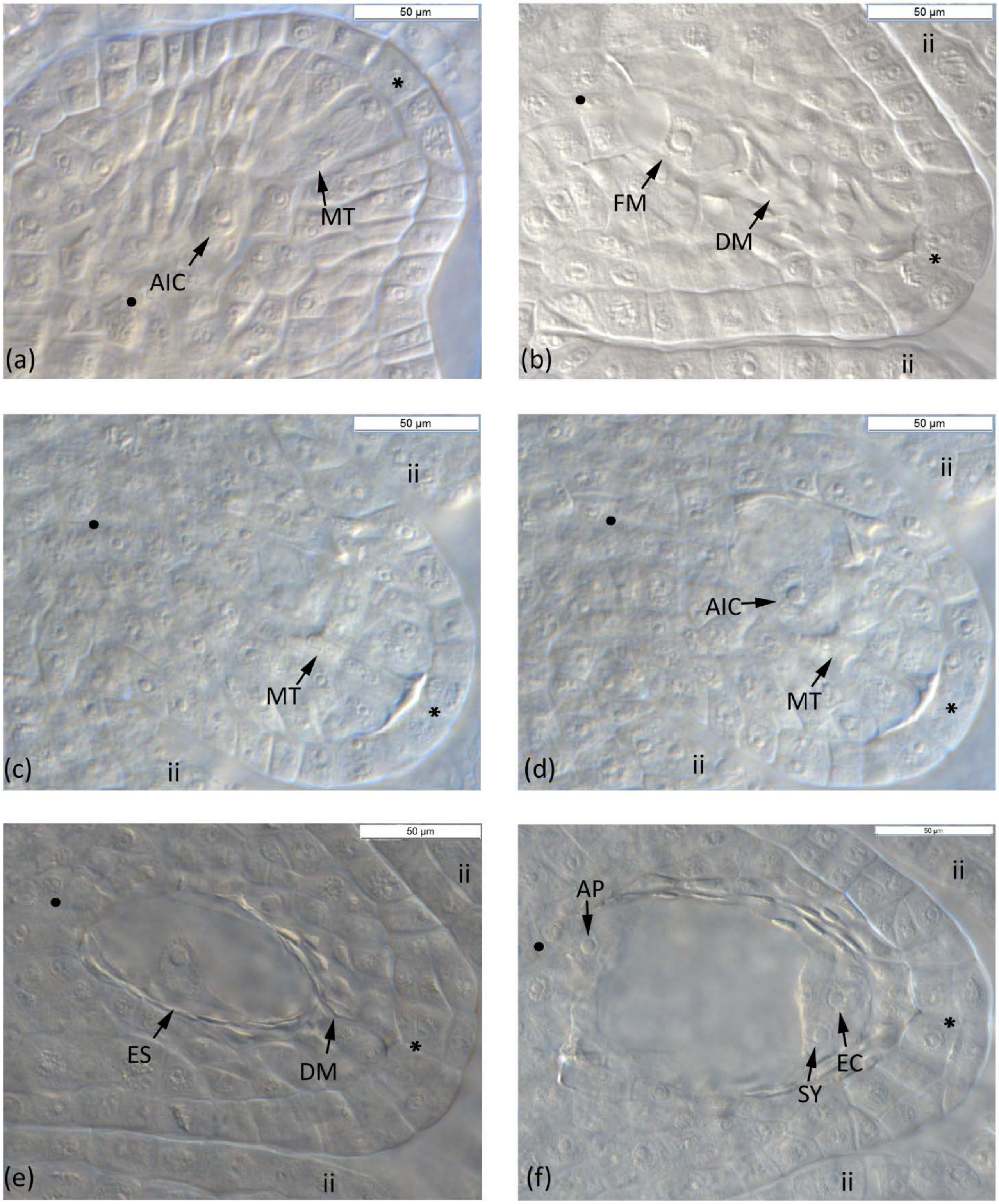

Supplementary Figure 1. Key reproductive stage of ovule development facultative apomictic diploid *R. carpaticola x notabilis*. **(a)** Asexual ovule with megaspores in meiotic division and an aposporous initial cell; **(b)** Sexual ovule with a functional megaspore enlarge within two vacuoles and three aborted megaspore at micropylar pole; **(c-d)** One asexual ovule in two different layers, with an aborted megaspore tetrad (c) and a big aposporous initial cell (d); **(e)** Sexual ovule with a young embryo sac at germline position (one nucleus visible and another in another optical layer); **(f)** Mature

embryo sac with egg cell and synergids at micropylar pole and antipodal cells at chalazal pole. Plant individual: **(a)** F3xJ6/25; **(b)** F10XF7/01; **(c-d)** F3xJ6/19; **(e-f)** J6XF3/23 . AIC, Aposporous Initial Cell; AP, Antipodal Cells; DM, degenerated megaspores; EC, Egg Cell; ES, Embryo Sac; FM, Functional Megaspore; ii, inner integument; MT, Megaspore Tetrad; SY, Synergid●, chalazal pole; *, micropylar pole. Scale bar: 50 µm.

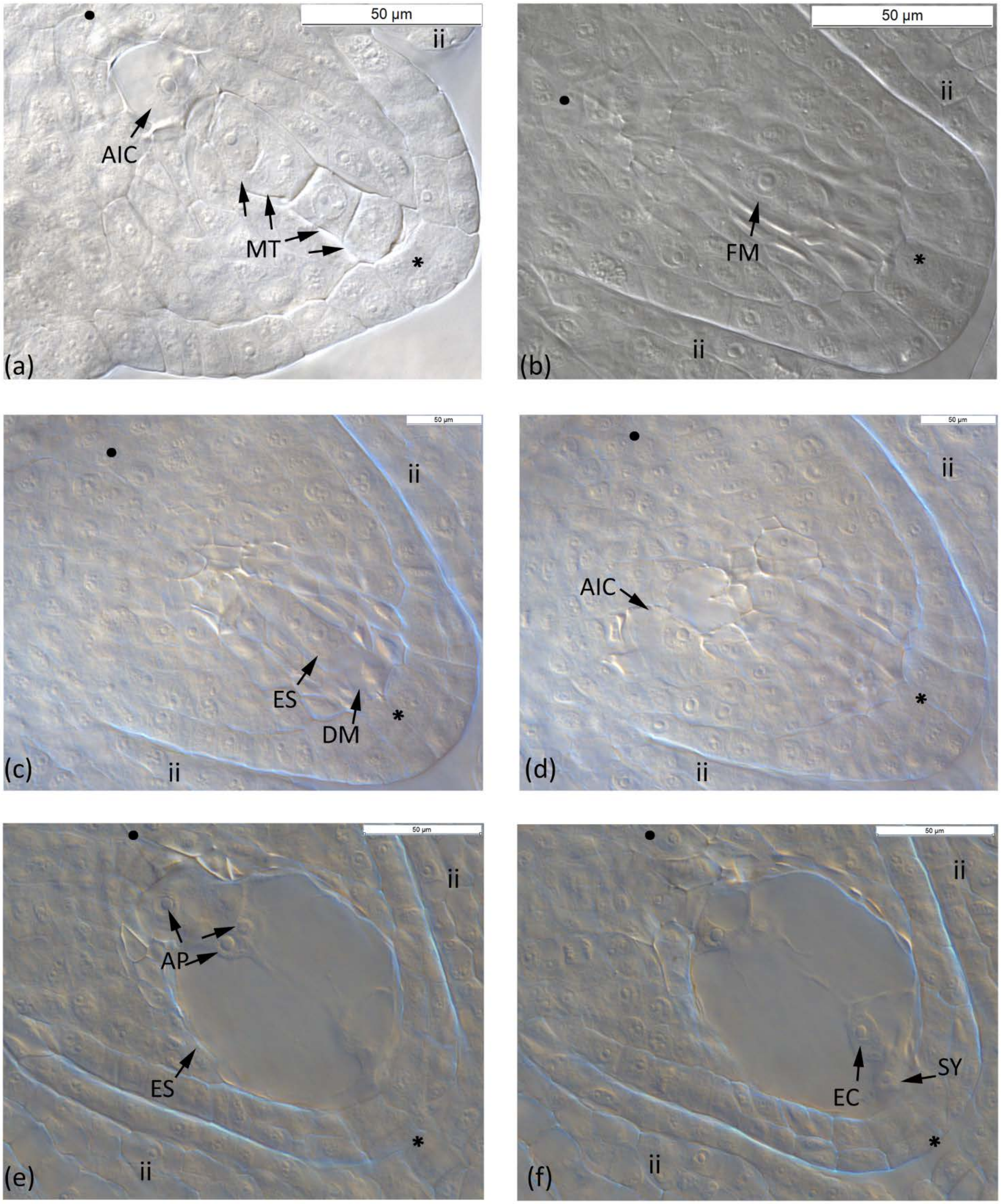

Supplementary Figure 2. Key reproductive stage of ovule development facultative apomictic tetraploid *Ranunculus variabilis*. **(a)** Asexual ovule with megaspore tetrads in alignment with an aposporous initial cell at chalazal pole; **(b)** Sexual ovule with an enlarged functional megaspore and degenerated meiotic products; **(c-d)** One asexual ovule in two different layers with a young (2-nucleate stage) meiotic embryo sac (c) and AIC (d); **(e-f)** One ovule in two different layers with a

mature embryo sac with an egg cell and two synergids near to micropylar pole and three antipodal cells at chalazal pole. Plant individual: **(a)** LH1406030B4-7; **(b)** LH1406030B4-19; **(c-d)** LH1406030B5-08; **(e-f)** LH1406030B5-08. AIC, Aposporous Initial Cell; AP, Antipodal Cells; DM, degenerated megaspores; EC, Egg Cell; ES, Embryo Sac; FM, Functional Megaspore; ii, inner integument; MT, Megaspore Tetrad; SY, Synergid; ●, chalazal pole; *, micropylar pole. Scale bar: 50 µm.

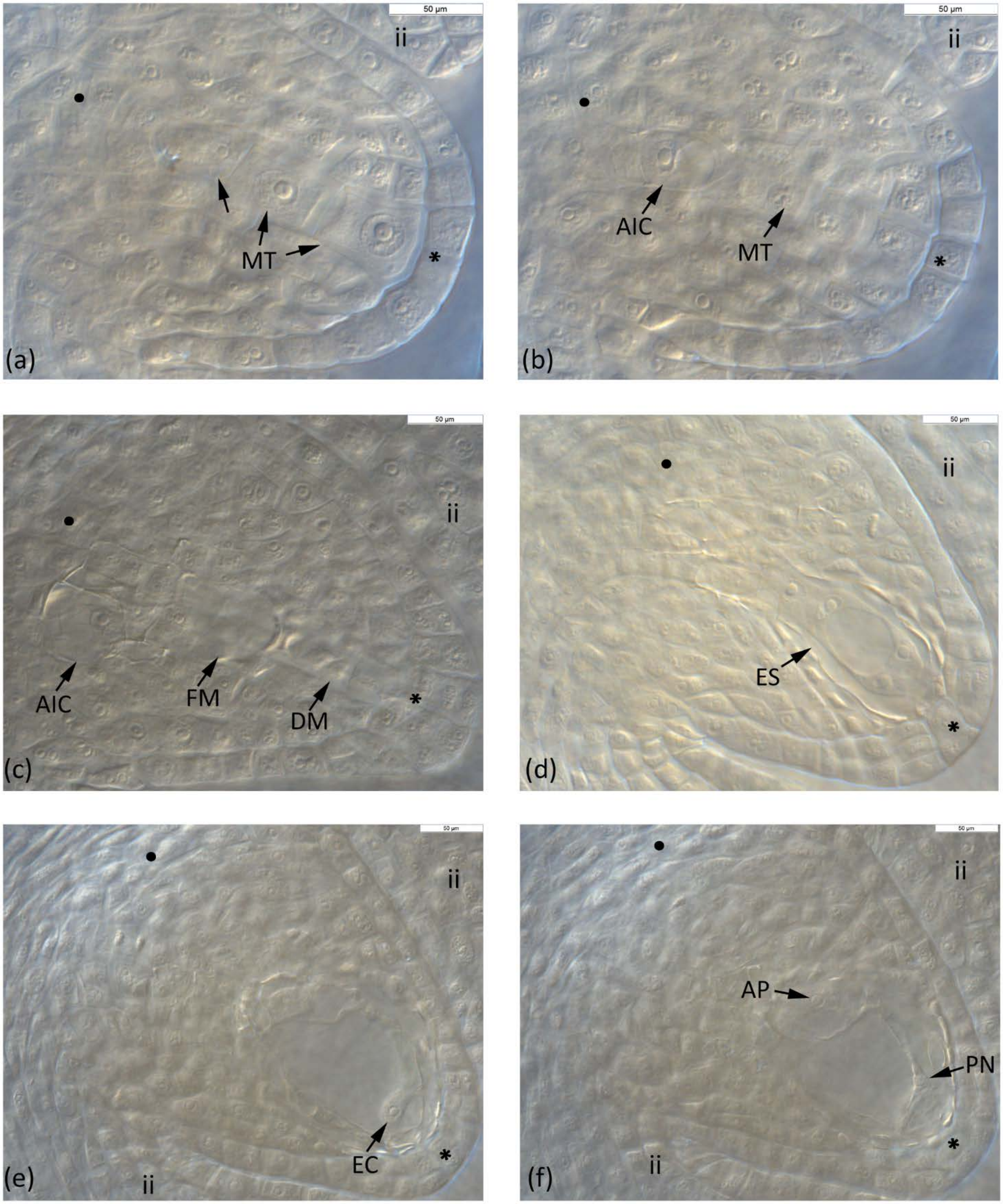

Supplementary Figure 3. Key reproductive stage of ovule development in facultative apomictic hexaploid *Ranunculus carpaticola* x *cassubicifolius.* **(a-b)** One asexual ovule in two different layers with megaspore tetrad cells in alignment (a) and an aposporous initial cell near chalazal pole (b); **(c)** Asexual ovule with functional megaspore and an AIC; **(d)** Ovule with young embryo sac after second nuclear division produced four nuclei; **(e-f)** One ovule in two different layers showing an embryo sac with an egg cell, polar nuclei, and Antipodal cells at chalazal pole; Plant individual: **(a-b)** 29/15-

3V2/04; **(c)** 29/15-1L3/01; **(d)** 8492/6-2/04; **(e-f)** 29/15-3V2/27. AIC, Aposporous Initial Cell; AP, Antipodal Cells; DM, degenerating megaspores; EC, Egg Cell; ES, Embryo Sac; ii, inner integument; MT, Megaspore Tetrad; PN, Polar Nuclei●, chalazal pole; *, micropylar pole. Scale bar: 50 µm.

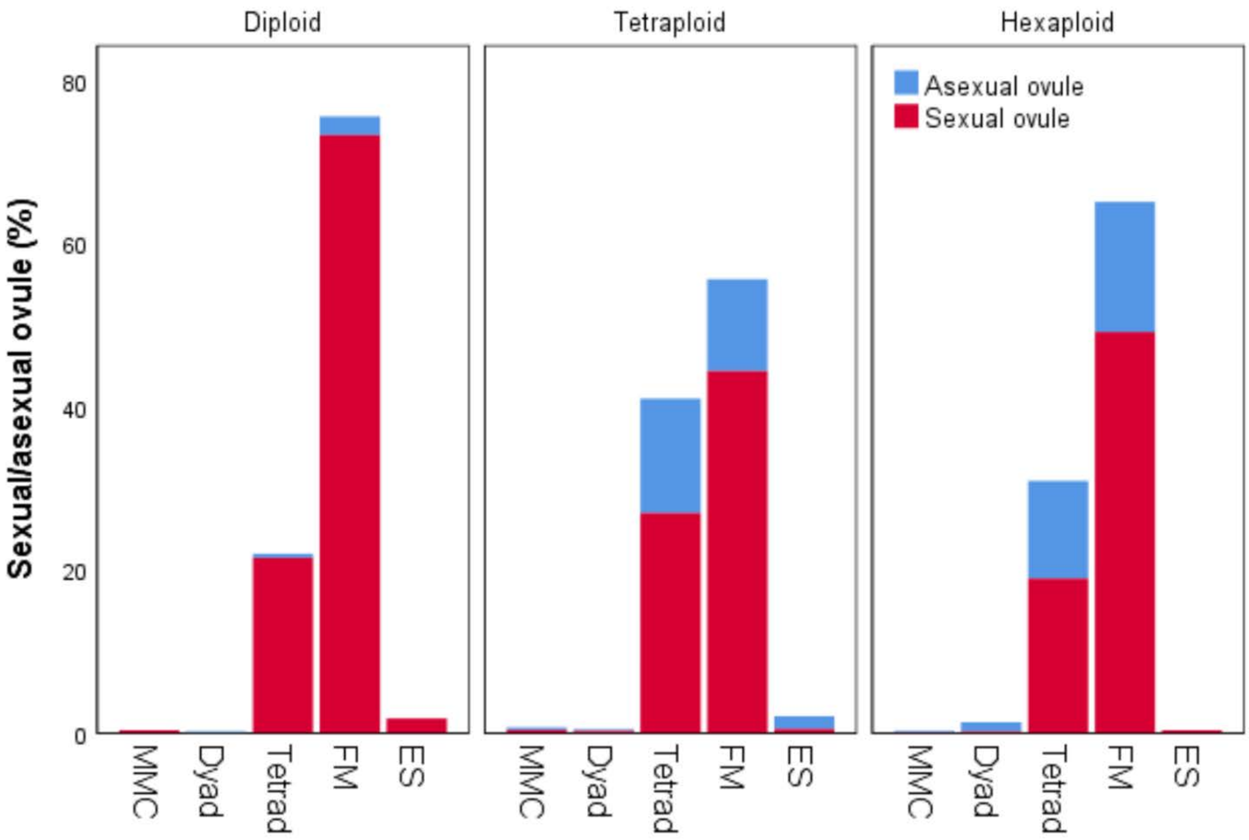

Supplementary Figure 4: Mean proportions of sexual and asexual ovules from each developmental stage in three cytotypes of the *R. auricomus* complex (both treatments pooled). MMC, Megaspore Mother Cell; Dyad, first meiotic product; Tetrad, completed meiosis has produced four megaspores; FM, Functional Megaspore (only the chalazal megaspore developed while the other three cells aborted); ES, young embryo sac.

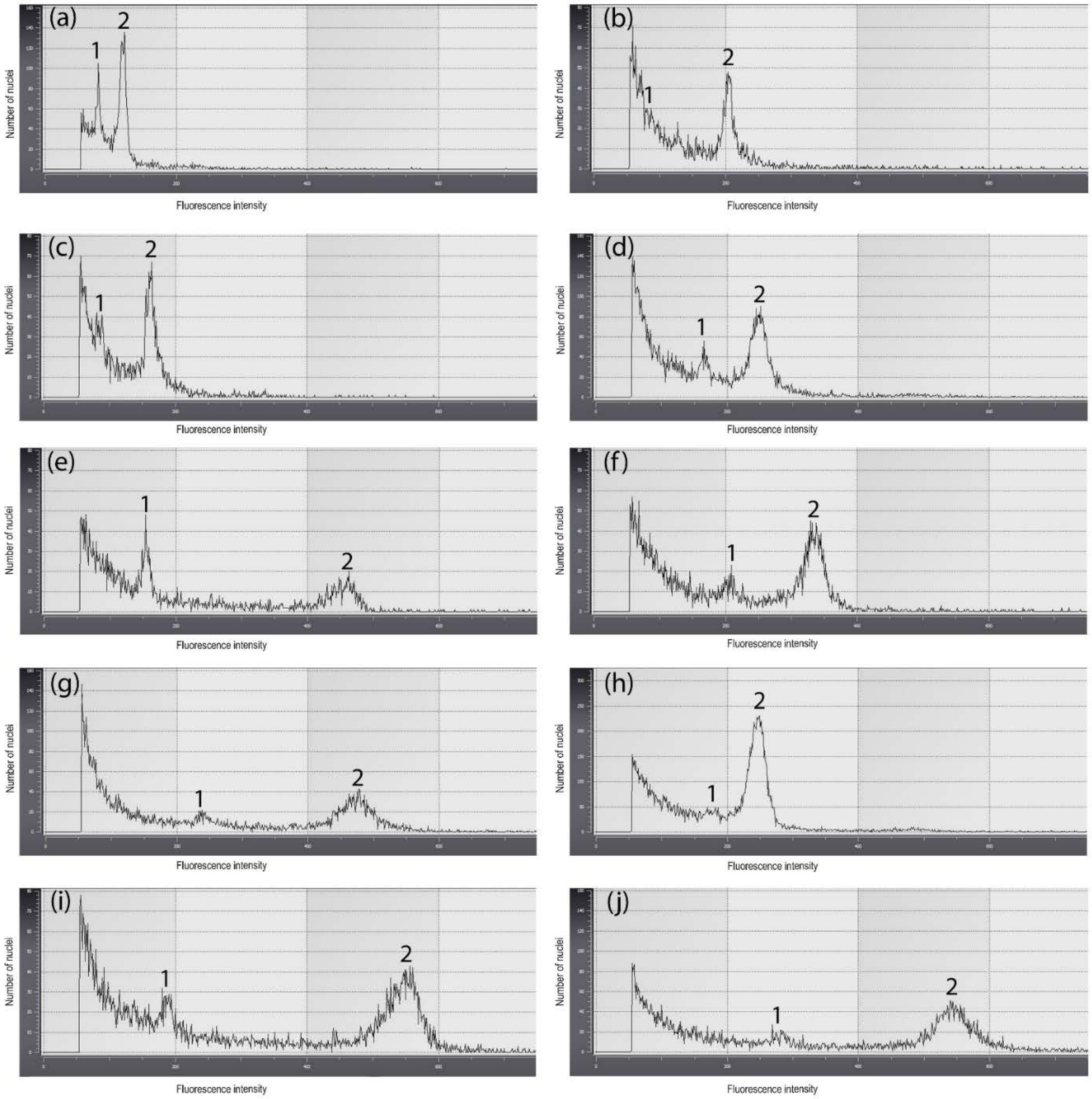

Supplementary Figure 5. Flow cytometry histograms of the *Ranunculus auricomus* complex. **(a)** diploid sexual seed; **(b)** diploid pseudogamous apomictic; **(c)** diploid autonomous apomictic; **(d)** tetraploid sexual; **(e)** tetraploid pseudogamous apomictic; **(f-g)** tetraploid BIII_hybrid; **(h)** hexaploid sexual; **(i)** hexaploid pseudogamous apomictic; **(j)** hexaploid BIII_hybrid. General peak labelling: 1 embryo peak, 2 endosperm peak. Plant individual: **(a)** J20xJ2/22; **(b)** F10xJ33/9; **(c)** F3xJ6/05; **(d)** LH1406030G1-16; **(e)** LH1406030B4-01; **(f)** LH1406030B2-07; **(g)** LH1406030B1-02; **(h-i)** 29/15-5K/31; **(j)** 29/15-6J/02.

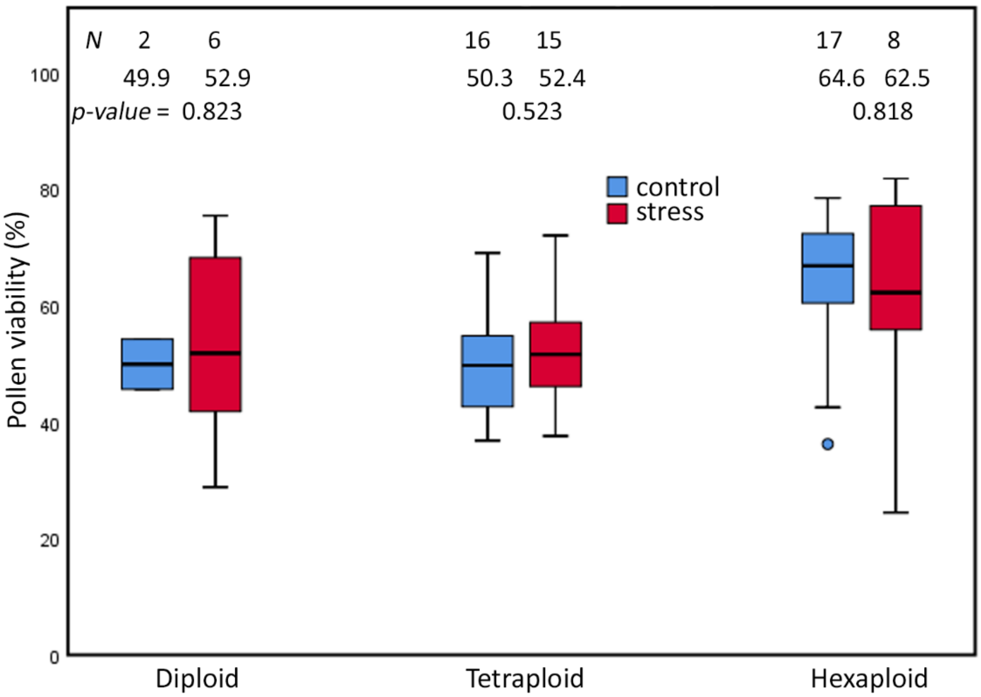

Supplementary Figure 6. Proportions of viable pollen in the *R. auricomus* complex plants grown in climatic chamber under prolonged photoperiod (stress) and shortened photoperiod (control). Mean values and statistical significance are given in figure. N = number of individuals. For the test statistic, see Suppl. Table S2.

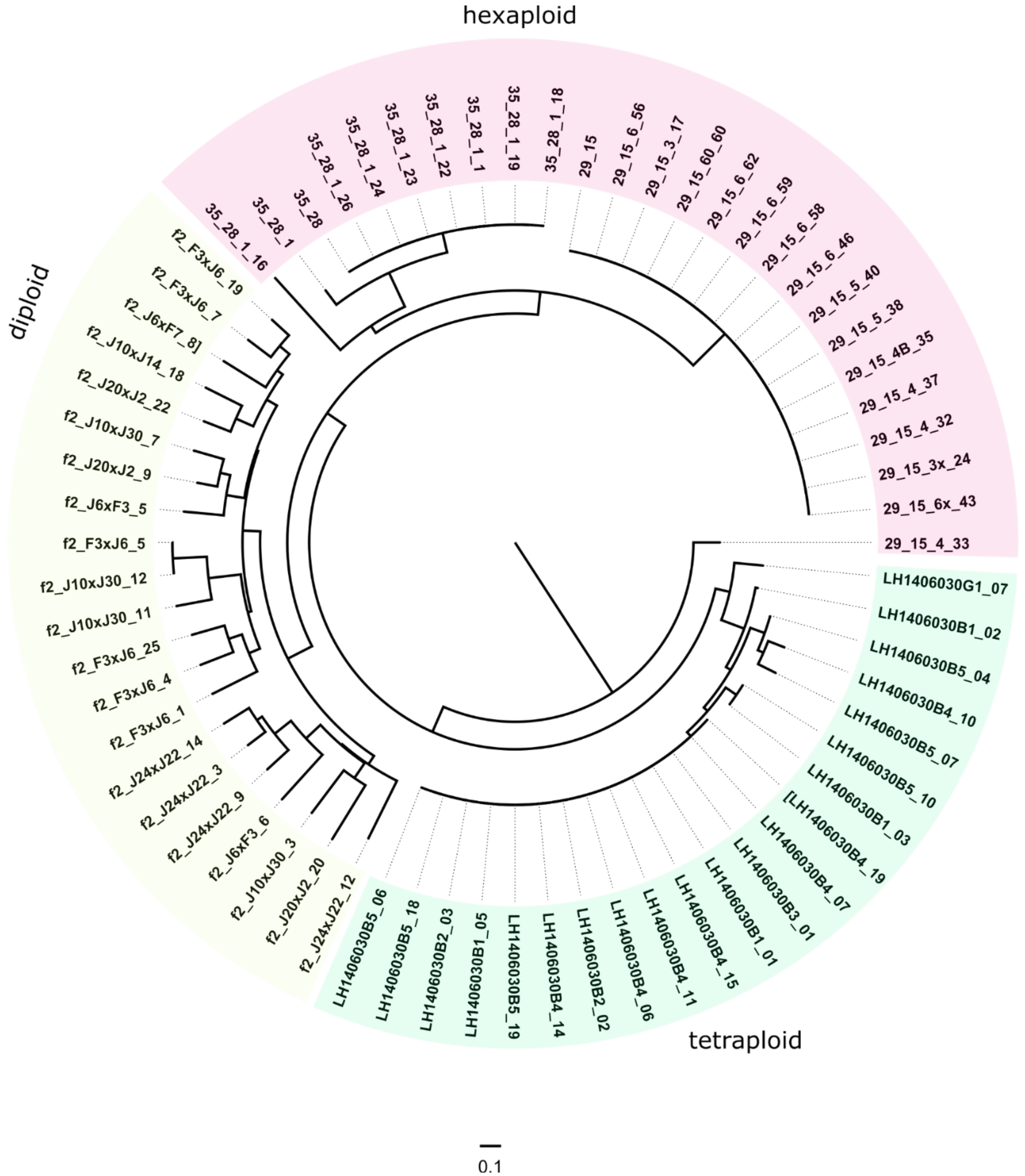

Supplementary Figure 7. Neighbor-joining tree derived from SSR data. *Ranunculus auricomus* complex from three different cytotypes and their clonal progeny were analyzed. Scale bar = no. of changes.

2 Supplementary Tables

Supplementary Table 1. Plant material list of *Ranunculus auricomus* complex from three different cytotypes. * indicated the plants used in different treatment in 2017 and 2018

**Species**	**2017**		**2018**	
	**Control**	**Stress**	**Control**	**Stress**
R. carpaticola x notabilis (2x)				
	F3XJ6/23	J10XJ2/22	F3xJ6/07	F3xJ6/04
		J24XJ22/10	F3xJ6/25	F3xJ6/05
		F3XJ6/10	J10xJ14/18	J10xJ30/12
		J10XJ30/12		J24xJ22/03
		J20XJ2/25		J24xJ22/23
		J24XJ22/18		F3xJ6/19
		J6XF3/23		F10xJ33/13
		F10XF7/01		F10xJ3/03
				J6xF3/06
				J24xJ22/09
Ranunculus variabilis (4x)				
	LH1406030B1-03*	LH1406030B1-01*	LH1406030B1-01*	LH1406030B1-03*
	LH1406030B5-18*	LH140603G1-07*	LH1406030B1-02*	LH1406030B1-04*
	LH1406030B2-02*	LH1406030B1-02*	LH1406030B2-03*	LH1406030B2-01
	LH1406030B4-06	LH1406030B1-05*	LH1406030B2-04	LH1406030B2-07
	LH1406030B4-10*	LH1406030B3-01*	LH1406030B3-01*	LH1406030B4-01
	LH1406030B4-11	LH1406030B2-03*	LH1406030B4-02	LH1406030B4-17
	LH1406030B4-14	LH1406030B4-7	LH1406030B4-08	LH1406030B4-20
	LH1406030B4-15	LH1406030B5-06	LH1406030B4-09	LH1406030B4-21
	LH1406030B4-19	LH1406030B5-07*	LH1406030B4-16	LH1406030B2-02*
	LH1406030B4-04	LH1406030B5-19*	LH1406030B4-18	LH1406030B5-08
	LH1406030B5-10*	LH1406030B4-07	LH1406030B5-07*	LH1406030B5-09
	LH1406030B4-10	LH1406030G1-07	LH1406030B5-12	LH1406030B5-10*
	LH1406030B5-04		LH1406030B5-16	LH1406030B5-13
			LH1406030B5-19*	LH1406030B5-18*
			LH140603G1-15	LH1406030B4-10*
			LH140603G1-07*	LH1406030G1-8
			LH4B005	LH1406030G1-16
				LH1406030G1-18
Ranunculus carpaticola x *cassubicifolius* (6x)				
	29/15-6J/12	8492/27-1B/03	29/15-3N/02	29/15-3V2/03
	29/15-1L3/01	29/15-3V2/22	29/15-3V2/27	29/15-1L3/02
		35/28-1/14	29/15-5K/05	29/15-1L3/11
		29/15-3V2/03	29/15-5K/09	29/15-3V2/04
			35/28-4*/13	29/15-5K/29
			35/28-3/61	35/28-1/16
			35/28-4*/19	29/15-5K/07
			29/15-5K/31	29/15-3N/22
			29/15-6J/02	
			8492/6-2/04	
			29/15-6J/12	
			29/15-1L3/01	

Supplementary Table 2. Statistical characteristic of the effect of light extension on the reproductive mode among ploidies of *Ranunculus auricomus* complex plants. P-values in bold indicate significances between light treatment.

	Diploid		Tetraploid		Hexaploid	
	**Control**	**Stress**	**Control**	**Stress**	**Control**	**Stress**
			**Proportion of sexual ovule**			
Median	80.37	99.26	57.90	80.29	52.61	70.36
Mean	80.37	100.00	58.97	84.34	56.94	80.00
SD	19.38	1.26	8.79	10.67	26.11	20.04
N	2	13	16	13	26	26
p-value	0.001		0.001		0.006	
			**Seed-set**			
Median	35.09	50.00	29.02	29.14	43.40	43.59
Mean	39.84	50.22	31.09	28.97	42.17	43.04
SD	23.02	14.89	9.66	7.75	12.65	10.92
N	5	16	17	20	16	9
p-value	0.300		0.459		0.880	
			**Reproduction mode**			
Median	100.00	100.00	2.50	0.00	10.00	3.60
Mean	100.00	98.74	6.59	3.88	9.66	5.08
SD	0.00	3.30	8.27	4.75	11.15	5.32
N	5	15	17	18	13	8
p-value	0.337		0.251		0.293	
			**Pollen viability**			
Median	49.90	51.80	49.70	51.60	11.73	62.20
Mean	49.90	52.97	50.33	52.44	64.62	60.70
SD	6.08	17.38	9.22	9.51	11.73	19.95
N	2	6	16	15	17	11
p-value	0.777		0.536		0.605	

Supplementary Table 3. Pairwise comparison with Tukey HSD tests were conducted to determine the simple main effect of photoperiod on the proportion of sexual ovules among ploidies.

**Treatment**	**Ploidy (I)**	**Ploidy (J)**	**Mean Difference (I-J)**	**Std. Error**	**p-value**	**95% Confidence Interval**	
						**Lower Bound**	**Upper Bound**
Stress	Diploid	Tetraploid	0.554*	0.094	0.000*	0.366	0.743
	Diploid	Hexaploid	0.677*	0.081	0.000*	0.514	0.84
	Tetraploid	Hexaploid	0.123	0.081	0.137	-0.040	0.286
Control	Diploid	Tetraploid	0.356	0.198	0.08	-0.044	0.756
	Diploid	Hexaploid	0.396*	0.194	0.047*	0.005	0.788
	Tetraploid	Hexaploid	0.040	0.084	0.633	-0.129	0.21

Based on observed means.

* The mean difference is significant at the 0.05 level.

Supplementary Table 4. Two-way ANOVAs were conducted to determine the interaction effect of photoperiod and ploidy level on the proportion of well-developed seeds.

**Source**	**Type III Sum of Squares**	**df**	**Mean Square**	**F**	**p-value**
Ploidy	0.445	2	0.222	11.167	0.000
Treatment	0.018	1	0.018	0.887	0.349
Ploidy x Treatment	0.050	2	0.025	1.246	0.293

a. R Squared = 0.307 (Adjusted R Squared = 0.262)

Supplementary Table 5. Multiple comparisons with Tukey HSD tests were conducted to determine the simple main effect of photoperiod on the proportion of well-developed seeds among ploidies

Ploidy (I)	Ploidy (J)	Mean Difference (I-J)	Std. Error	p-value	95% Confidence Interval	
					Lower Bound	Upper Bound
Diploid	Tetraploid	0.173*	0.043	0.000	0.088	0.259
Diploid	Hexaploid	0.036	0.047	0.445	-0.057	0.129
Tetraploid	Hexaploid	-0.138*	0.037	0.000	-0.212	-0.063

Based on observed means.

* The mean difference is significant at the 0.05 level.

Supplementary Table 6. P-values for the Mann-Whitney U-test were conducted to determine the interaction effect of photoperiod and ploidy level on the proportion of sexual seeds.

	**Diploid**	**Tetraploid**	**Hexaploid**
Mann-Whitney U	30	118.5	47
Wilcoxon W	150	271.5	83
Z	-1.053	-0.678	-0.366
Asymp. Sig. (2-tailed)	0.292	0.497	0.714
Exact Sig. [2*(1-tailed Sig.)]	0.553c	0.533	0.750

Supplementary Table 7. Multiple comparisons with Tukey HSD tests were conducted to determine the simple main effect of photoperiod on the proportion of viable pollen among ploidies

**Ploidy (I)**	**Ploidy (J)**	**Mean Difference (I-J)**	**Std. Error**	**p-value**	**95% Confidence Interval**	
					**Lower Bound**	**Upper Bound**
Diploid	Tetraploid	0.015	0.063	0.968	-0.136	0.167
Diploid	Hexaploid	-0.015	0.063	0.968	-0.167	0.136
Tetraploid	Hexaploid	-0.152*	0.042	0.001	-0.253	-0.053

Based on observed means.

* The mean difference is significant at the 0.05 level.
